# Preparation of Poly(acrylate)/Poly(diallyldimethylammonium) Coacervates without Small Counterions and Their Phase Behavior upon Salt Addition towards Poly-Ions Segregation

**DOI:** 10.3390/polym13142259

**Published:** 2021-07-09

**Authors:** Marcos Vinícius Aquino Queirós, Watson Loh

**Affiliations:** Institute of Chemistry, University of Campinas (UNICAMP), P.O. Box 6154, Campinas 13083-970, SP, Brazil; m212103@dac.unicamp.br

**Keywords:** polyelectrolyte complexes, coacervation, associative phase separation, segregative phase separation, small counterions

## Abstract

In this work, we report the phase behavior of polyelectrolyte complex coacervates (PECs) of poly(acrylate) (PA^−^) and poly(diallyldimethylammonium) (PDADMA^+^) in the presence of inorganic salts. Titrations of the polyelectrolytes in their acidic and alkaline forms were performed to obtain the coacervates in the absence of their small counterions. This approach was previously applied to the preparation of polymer–surfactant complexes, and we demonstrate that it also succeeded in producing complexes free of small counterions with a low extent of Hofmann elimination. For phase behavior studies, two different molar masses of poly(acrylate) and two different salts were employed over a wide concentration range. It was possible to define the regions at which associative and segregative phase separation take place. The latter one was exploited in more details because the segregation phenomenon in mixtures of oppositely charged polyelectrolytes is scarcely reported. Phase composition analyses showed that there is a strong segregation for both PA^−^ and PDADMA^+^, who are accompanied by their small counterions. These results demonstrate that the occurrence of poly-ion segregation in these mixtures depends on the anion involved: in this case, it was observed with NaCl, but not with Na_2_SO_4_.

## 1. Introduction

Electrostatic complexes are formed through the associative phase separation between oppositely charged macro-ions, for example, natural or synthetic polyelectrolytes, surfactant aggregates, or inorganic nanoparticles. This association gives rise to a phase that is enriched in the charged species. Polyelectrolyte complexes (PECs) is the term given to the colloidal aggregates, dense liquid phase and solid phase that result from the association between oppositely charged polyelectrolytes. When this phase contains high amounts of water, it behaves like a liquid and it is commonly named as coacervate [1,2]. Their formation involves at least four ionic species: a polycation (Pol^+^), a polyanion (Pol^−^), a small cation (M^+^), and a small anion (A^−^). The complexation driving force is proposed to be almost entirely entropic, due to the release of water molecules and small counterions [3,4]. The PECs have been studied for many years and several studies highlight their potential to be used as porous membranes [5], emulsion stabilizers [6], self-healable materials [7], among others. PEC formation has been even observed in biological systems, composing many cellular compartments that are known as membraneless organelles [8]. Several studies exploit variations in pH [9], temperature [10], molar mass [2] and the chemical nature of the polyelectrolytes [11] as well as the salt addition in order to understand what implications they have on the properties of the PECs.

Salt content is, by far, the most studied factor and it plays an important role in controlling the thermodynamic [12,13,14], morphological [2,15,16], and mechanical [17] properties of the PECs. The electrostatic pairing not only takes place between the charged units from the polymer chains, but also among the small counterions and the poly-ion ionic species [18]. The addition of an electrolyte causes charge screening and increases the number of poly-ion/small counterion pairs. Further addition of salt leads to the total dissolution of the PEC, and its poly-ion chains remain free in the solution. These phenomena, restricted to the associative phase separation, are better visualized through their phase diagrams, which have been described by several experimental and theoretical studies, as we summarize below.

A commonly used representation for the phase behavior of PECs is by plotting the concentrations of the salt and poly-ion charged units into a rectangular phase diagram, in which the associative phase separation appears as a binodal curve [19,20]. In this representation, it is possible to estimate the strength/stability of a PEC through the salt concentration needed to dissolve it, at a certain polymer concentration [2,21]. Ternary phase diagrams could also be designed by taking the oppositely charged polyelectrolytes and water as a three-component mixture. Such a plane, as previously described for polymer-surfactant systems, is referred to as the conventional mixing plane [22]. Considering salt addition, the ternary phase diagram could be better represented if a pre-existent poly-ion complex, that is, the PEC free of small counterions, water, and the simple salt are taken as the three components. This representation was early referred to as the alternative mixing plane [22], and is actually a pseudo three-component phase diagram because it results from a true four component-system, formed by the combination of the four charged species and water. One example of the use of this phase diagram was reported for a mixture of the poly(styrene sulfonate)/poly(diallyldimethylammonium) complex with water and KBr [23]. In order to obtain the ‘pure complex’, this PEC was extruded and annealed in salty solution followed by soaking in pure water for complete small ions removal.

Another approach, initially developed for the investigation of polyelectrolyte–surfactant complexes [24], consists in titrating the acidic and alkaline forms of the ionic components, either surfactants or polyelectrolytes. Such an approach has allowed the study of the phase behavior of binary/ternary mixtures of these polymer–surfactant complexes with several other additives, including n-alcohols [25], oils [26], and salts [27]. The present study aims to extend this approach to the study of the phase behavior of PEC mixtures with water and inorganic salts. The complexes were composed of poly(acrylate) (PA^−^), which is the conjugate base of poly(acrylic acid), and poly(diallyldimethylammonium) (PDADMA^+^), which was converted to its hydroxide form through ion exchange. Two parameters were varied: the poly(acrylate) molar mass (2.0 kDa and 100 kDa), and the type of salt added (NaCl and Na_2_SO_4_). The present work goes beyond the associative phase separation by varying the salt concentration in a range which made it possible to observe a second phase separation, identified as segregative. This region was described in terms of their phase composition, on which, to the best of our knowledge, there are no reports for mixtures of oppositely charged polymers.

## 2. Materials and Methods

### 2.1. Materials

Poly(acrylic acid) (PAA, M_w_ of 2.0 kDa (PA_2k_) and 100 kDa (PA_100k_)), poly(diallyldimethylammonium chloride) (PDADMAC, M_w_ in the range of 100–200 kDa), sodium hydroxide (NaOH), sodium chloride (NaCl), sodium sulfate (Na_2_SO_4_) and a Dowex^®^ Monosphere^®^ 550A (OH) ion exchange resin were obtained from Sigma-Aldrich Brasil (Cotia, Brazil). These chemicals were used as received.

### 2.2. Preparation of the PECs through Acid–Base Titration

This procedure follows the method that Svensson and coworkers initially proposed for polymer–surfactant complex preparation [24]. PDADMAC solution (100 mL, 0.32 mol L^−1^ in charge) was prepared in deionized water and left in contact with an anion exchange resin previously activated with NaOH 1 mol L^−1^. Ion exchange took place in three steps: the solution was placed in one fraction of the resin for two hours, transferred to another fraction for another hour followed by a third fraction for one more hour. All steps occurred under magnetic stirring with a total contact time of 4 h. The polycation on its basic form is called PDADMAOH.

**Figure 1:**
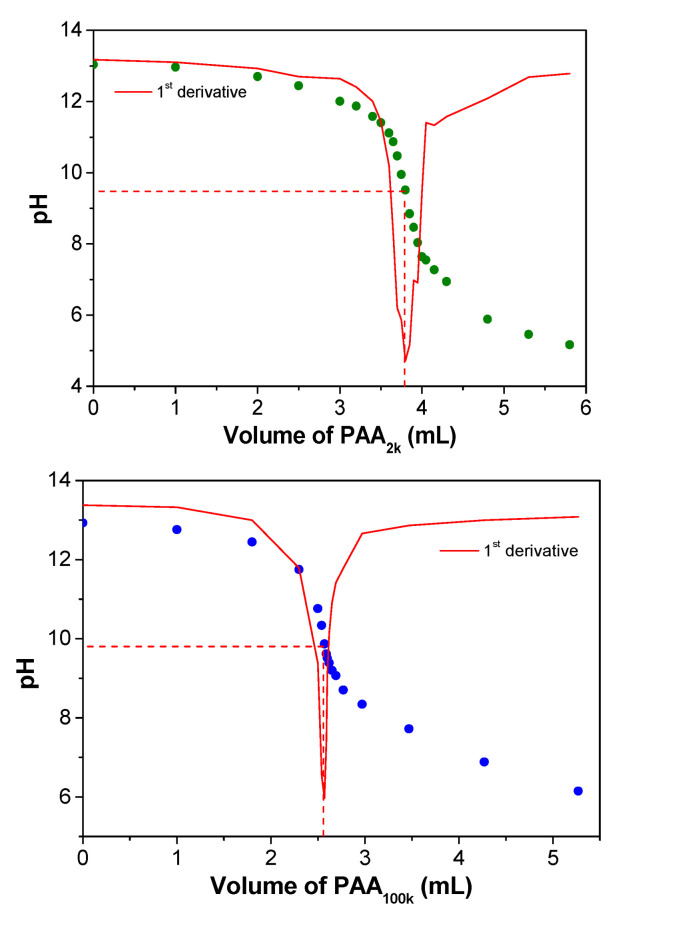
Titration curves of 20 mL of a PDADMAOH solution (concentration after ion exchange is around 50–70 mmol L^−1^) with addition of a PAA solution (2.0 and 100 kDa; 0.35 mol L^−1^ in repeat units).

After ion exchange, 20 mL of the PDADMAOH solution is then titrated with PA_2k_ or PA_100k_ (0.35 mol L^−1^ in repeat units) by using the titrator model 848 Titrino plus from Metrohm (Herisau, Switzerland), equipped with a pH electrode model 6.0262.100. Coacervation takes place according to the acid–base reaction, described in Equation (1) with resulting titration curves shown in Figure 1. Then, PAA solution is added to the remaining PDADMAOH solution until achievement of the pH of the equivalence point. At this point, the charge stoichiometry is taken as 1:1. The resulting coacervate is left with its supernatant for at least 24 h. Then, the supernatant is removed and the coacervate is collected and centrifuged to better separate it from the residual supernatant. The complexes were named PDADMAPA_x_, where x can be 2k or 100k, depending on the molar mass of PA^−^.

PDADMA^+^OH^−^(aq) + PAA-H(aq) → PDADMA^+^PA^−^(s) + H_2_O(l) (1)

### 2.3. Assessment of Hofmann Elimination Extent

**Scheme 1:**
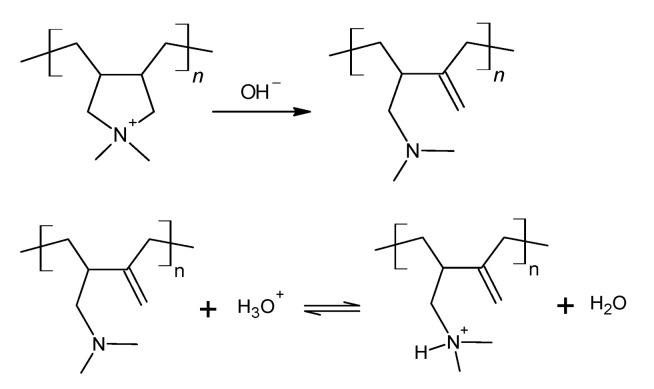
Reactions describing the Hofmann elimination in a PDADMA^+^ chain followed by the protonation of the tertiary amine as the pH decreases.

A Hofmann elimination reaction takes place on quaternary amines under alkaline conditions. It is characterized by the breakage of the C-N bond and the formation of a C=C double bond. In this work, PDADMA^+^ contains a quaternary amine in a five-member ring. This reaction occurs according to Scheme 1, at which the C-N bond breakage leaves the nitrogen attached to one carbon atom and makes it available to act as a weak base. Its protonation is also shown in Scheme 1. Based on that information, it is possible to quantify the extent of Hofmann elimination through pH titrations of the alkaline polycation solution with a standard acidic solution. We expect to observe two inflection points, the first one corresponding to the neutralization of the hydroxide ions from the ion exchange, and the second one related to the protonation of the tertiary amine resulting from the Hofmann elimination reaction. The inflexion points provide the information needed to calculate the extent (E/mol%) of this reaction according to Equation (2). Here, V_1_ and V_2_ stand for the volume of HCl solution necessary to reach the first and second inflexion points, respectively.

E = (V_2_ − V_1_) × 100%/V_2_ (2)

**Figure 2:**
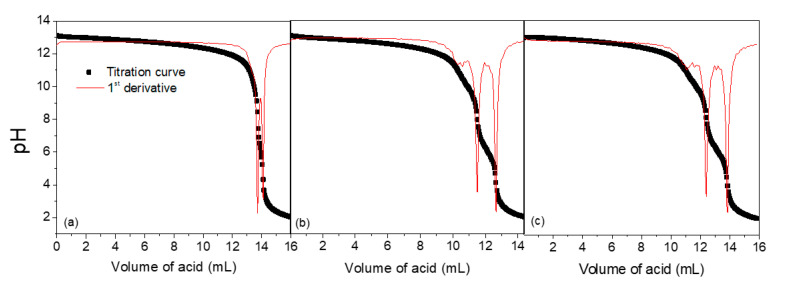
Titration curves of 20 mL of PDADMAOH solutions with 0.1 mol L^−1^ HCl solutions (**a**) immediately; (**b**) 24 h and (**c**) 48 h after their removal from the ion-exchange resin.

The data obtained from the titrations are exhibited in Table 1 and Figure 2. The extent of the reaction is small as the polycation solution is just removed from the resin. However, the reaction becomes progressively higher for a period of hours after the removal. For this reason, we used the solutions to prepare the complexes as soon as they were removed from the resin. It is worth stressing that this constitutes a simple and reliable method to quantify the extent of this elimination in aqueous media.

### 2.4. Determination of Chloride in the Bulk Coacervates

Appropriate amounts of the coacervates were weighed and dissolved in 1.0 mol L^−1^ NaNO_3_ solution to break the coacervates and to release all the ions. Potentiometric titrations were performed with standard AgNO_3_ solutions by using the titrator model 848 Titrino plus from Metrohm (Herisau, Switzerland) equipped with a silver electrode model 6.0430.100 Ag Titrode. The titrations were conducted in triplicate of independent samples.

### 2.5. Determination of the Water Content into the Bulk Coacervates

Appropriate amounts of the coacervates were weighed into glass flasks that were placed in an oven at 60 °C for several days until there was no significant variation in their masses. The mass difference resulting from drying corresponds to the water content in the coacervates.

### 2.6. Preparation of PDADMAPA_2k_ through Direct Mixing

Another PEC (PDADMAPA_2k_) was prepared through the direct mixing of a PA^−^ solution with a PDADMA^+^ solution. Prior to mixing, the pH of these solutions was adjusted to pH 10 by adding NaOH to keep poly(acrylate) fully charged. Appropriate amounts of both polyelectrolytes were mixed in order to obtain a 1:1 stoichiometric complex coacervate. After its preparation, this PEC was submitted to the same procedures described in the Section 2.4 and Section 2.5 for comparison with the PECs prepared through acid–base titration.

### 2.7. Preparation of the Mixtures Containing PEC, Water, and Salt

Appropriate amounts of PDADMAPA, water and salts (NaCl and Na_2_SO_4_) were weighed and mixed in a glass vial. Homogenization of the mixtures was facilitated with the use of a vortex. The previously determined amount of water in the PECs was taken into account for the calculation of its global mass fraction.

In order to define the phase separation boundaries, three types of additions were performed: small increments of dried solid salt, water or salt solution, were added until a visual cloudiness occurred or disappeared. Such a phenomenon was carefully verified by visual inspection after each increment, producing a resolution ≤ 0.5 wt %. Some points required a few days to ensure that equilibrium was achieved because of their high viscosity.

### 2.8. Determination of the Phase Composition in the Segregative Phase Separation

**Figure 3:**
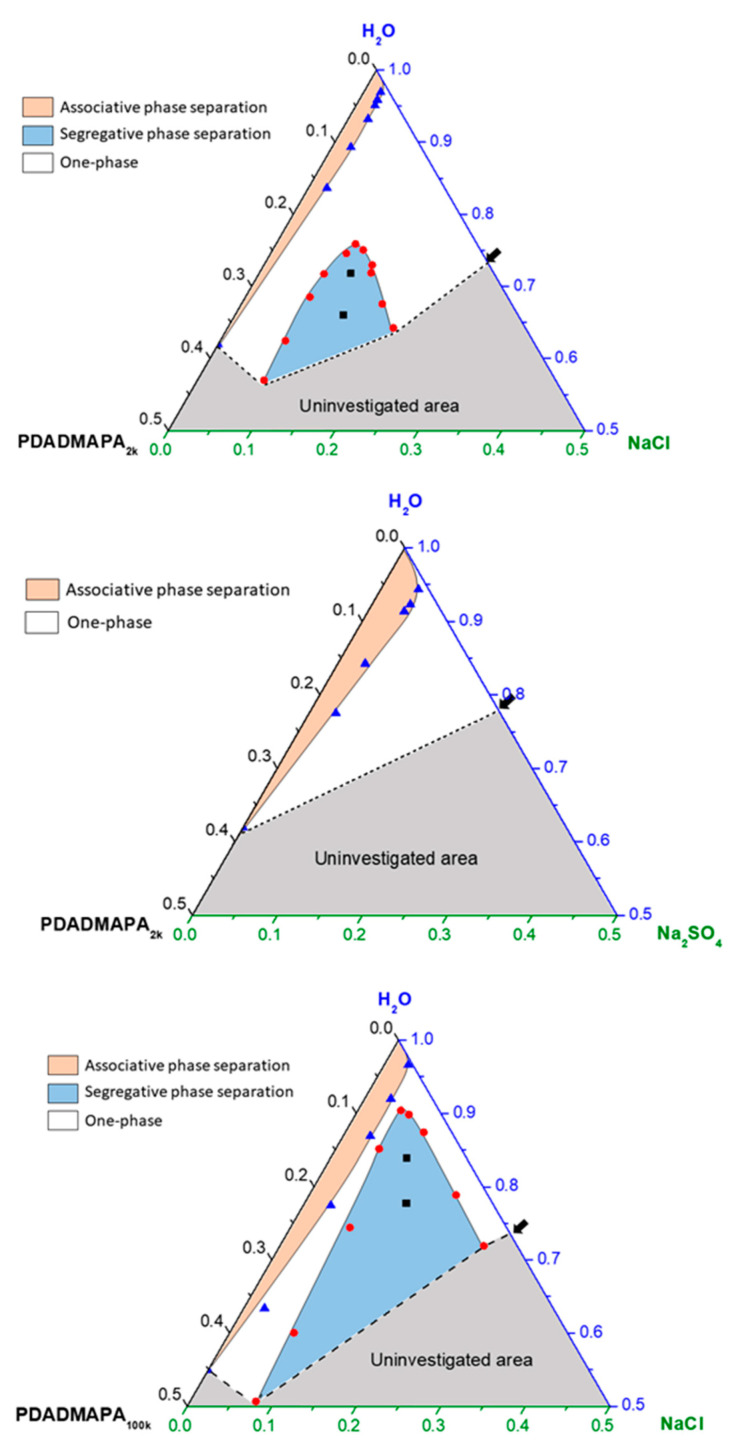
Pseudo-ternary phase diagrams of mixtures containing PDADMAPA, water and salts. Experimental points are indicated for (▲) associative and (●) segregative phase separation regions. The black squares (■) denote the global composition for the systems whose phases were analyzed. The arrows indicate the solubility of the salts in water. The axes are expressed in mass fraction.

The following procedures were employed for the quantification of the five different species (water, Na^+^, Cl^−^, PDADMA^+^, and PA^−^) in the segregative phase separation region. The samples were prepared according to the procedure described in the last section at the compositions indicated in Figure 3. After the preparation, they were centrifuged at 25 °C until displaying completely clear phases. The following analyses were conducted for each one of these separated phases. All of the measurements were carried out in triplicate, using independent samples.

PA^−^ was determined from pH titrations. Appropriate amounts of each phase were weighed and diluted in 0.5 mol L^−1^ NaCl solutions for the samples of PDADAMPA_2k_ and 1.0 mol L^−1^ KBr solutions for those containing PADAMAPA_100k_. Thereafter, the pH of the solutions was adjusted to pH 12 using concentrated NaOH solution. Then, the resulting solutions were titrated with standard hydrochloric acid solutions. Two inflection points were determined, where the first one corresponds to the neutralization of NaOH and the second one to the neutralization of the carboxylate groups from poly(acrylate). The total concentration of carboxylates is determined by the difference between these two volumes. One example of these titrations is shown in Figure S1 in the Supporting Information.

Similar to what was done in the bulk phase of the PECs, appropriate amounts of each phase were weighed and dissolved in a 0.5 mol L^−1^ NaNO_3_ solution. After that, potentiometric titrations were conducted with standard AgNO_3_ solutions (see Section 2.4 above).

The content of Na^+^ in the phases were determined by inductively coupled plasma optical emission spectrometry (ICP-OES) measurements, using an equipment from PerkinElmer, model Optima 3000 (Waltham, MA, USA). For that, appropriate amounts of the samples were weighed and diluted in a 1 wt % HNO_3_ solution.

Water and PDADMA^+^ contents were determined by thermogravimetric analyses (TGA). Each one of the phases was collected and analyzed in an equipment from TA Instruments, model TGA55 Discovery (New Castle, DE, USA). The analyses were conducted in a temperature range of 25–800 °C under a nitrogen atmosphere with a heating rate of 10 °C/min. Water content could be measured directly from the thermograms because of the first mass loss event that corresponds to water evaporation. The second thermal event corresponds to polymer decomposition, it not being possible to separate PDADMA^+^ from PA^−^. Therefore, PDADMA^+^ concentrations from the upper phase were calculated by taking the difference between the mass loss in this second event and the concentrations of the PA^−^ determined by pH titrations. For most of the bottom phases, the amount of PDADMA^+^ is too low to be accurately separated from the amount of PA^−^. Therefore, they were estimated from the mass balance between the amount of the polycation in the upper phase and its global amount in the mixture. More details regarding these analyses are presented in the Supporting Information, including examples of the analyses of the phases and the pure polymers solutions in Figures S2 and S3, respectively.

## 3. Results

### 3.1. Contents of Water and Chloride in the PECs

Table 2 presents the molar ratio between the amount of residual chloride ions and the number of positive charges from PDADMA^+^ in the pure PEC samples. The chloride ions are the counterions of PDADMA^+^ before ion exchange. The analysis was done in order to evaluate the performance of the ion exchange, because this work intends to prepare PECs as free as possible of small counterions. The results in Table 2 show that, for the complex PDADMAPA_2k_, the molar ratio between the remaining chloride ions and positive charges is 6:1000. Just for comparison, another PEC was prepared through the direct mixing of the polyelectrolytes (see Section 2.6 above), and its chloride content was measured (see Table 2). This PEC presents a higher chloride to positive PDADMA^+^ charges molar ratio (130:1000), also associated with an increase in its water content. These data suggest that the acid–base titration approach ensures a high performance for counterion removal in such a way that no further procedures are required to remove salt from the coacervates.

In addition, this approach brings a question regarding the driving force behind the electrostatic complexation in the absence of counterion release. Although there is an enthalpic contribution, in general the values of complexation enthalpies measured by calorimetric titrations are small [12,28]. In addition, Fu and Schlenoff [12] demonstrated that the changes in enthalpy upon the association between oppositely charged polyelectrolytes occur due to changes in water perturbation. Those authors also pointed out that the entropy contains the major contribution for complexation free energy over a wide range of salt concentrations. This entropic gain is commonly attributed to the release of counterions and water molecules [3,4]. It has been reported by Park and coworkers that water release may play a major role than the counterion release [29]. Based on that information, the accomplished preparation of these electrostatic complexes via acid–base titration supports that the release of water molecules must be more relevant to the entropic gain than the counterion release.

Table 2 also reveals that there is a difference between the water content in the different PECs, with PDAMAPA_2k_ presenting a higher value (61.9 wt %) than PDAMAPA_100k_ (54.9 wt %). Both PECs behave as viscous liquids, meaning that they could be described as coacervates [1,2]. Their high water contents (above 50 wt %) agree with those reported for poly(acrylate) complexes [11]. However, we emphasize that complexes prepared with poly(acrylate) do not always undergo spontaneous coacervation. The type of polycation also determines the morphology of their resulting PECs. The mixture of poly(acrylate) with poly(allylamine hydrochloride), for example, phase separates as a solid-like precipitate at lower salt concentrations [30]. The difference in the morphologies of the PECs could result from the different strength of the complexes. The strongest ones usually phase separate as solid-like precipitates [11]. Even PA^−^ and PDADMA^+^ can present this morphology at lower pHs. In order to induce a morphological transition from precipitate to coacervate, the pH [6,31] as well as the salt concentration [15,16] can be increased. In the present work, the PECs phase separated as liquid-like coacervates because they were prepared with no other counterions at a relatively high pH (the equivalence points are in the range of 9–10, see Figure 1).

### 3.2. Phase Behavior in Mixtures Containing PDADMAPA, Water and Salt

The following results describe the phase behavior of the complexes PDADMAPA_2k_ and PDADMAPA_100k_ upon NaCl addition. The phase behavior of the PEC with the smaller poly(acrylate) was also studied upon Na_2_SO_4_ addition. Their phase behavior could be represented as a pseudo three-component phase diagram, as depicted in Figure 3.

The mixtures containing PDADMAPA (2k and 100k), water and NaCl present two different regions of phase separation. The first one, next to the binary line PDADMAPA-water, denotes the associative phase separation region. Its major feature is the presence of both polymers in the same phase, which is in equilibrium with an aqueous diluted phase. Looking at the whole diagram, this region seems quite thin when it is compared to the other one. The latter is associated to a segregative phase separation, in which it is expected that each one of the different phases will be enriched in one of the poly-ions. The details regarding each one of these regions will be discussed in the next sections.

#### 3.2.1. Associative Phase Separation

**Figure 4:**
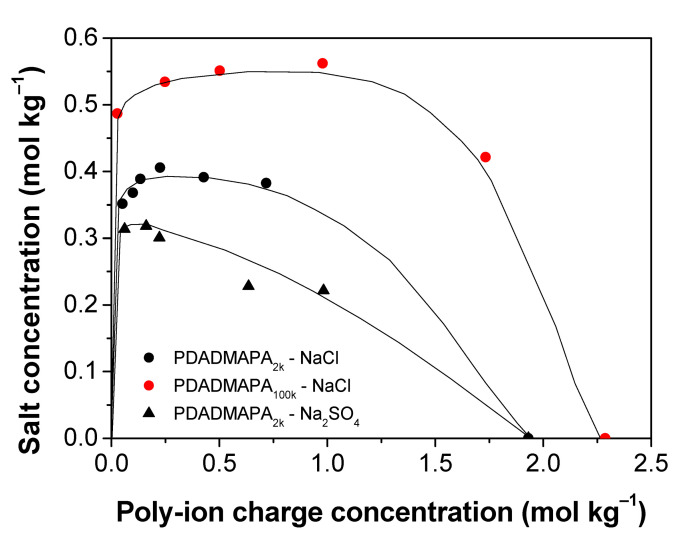
Phase diagrams for the systems containing PDADMAPA and different salts in the associative phase separation region. The lines are only guide to the eye. Phase separation occurs under each line. Above them, the mixtures are monophasic.

The effects of polymer molar mass and of the type of salt on this phase separation region can be more easily visualized in the rectangular phase diagram displayed in Figure 4. The charge concentration of the salts (mol kg^−1^) is expressed as a function of the charge concentration of the complex ion pairs. The resulting binodals allow us to estimate the stability of the PECs directly through the maximum salt concentration required to dissolve them.

In this respect, the PEC with the larger molar mass (PA_100k_) is more stable than the one with the smaller molar mass (PA_2k_). Several earlier studies also report that the increase in the molar mass of the poly-ions increases the stability of their PECs with respect to salt addition [2,12,19,32]. As the polymer chains become longer, their translational and configurational entropy per mass of added polymer decrease, resulting in an expansion of the two-phase region.

Regarding the chemical nature of the salts, the complex PDADMAPA_2k_ is more stable towards addition of NaCl than Na_2_SO_4_. This behavior was also observed for poly(acrylic acid)/poly(allylamine) complexes [33]. This could be explained in terms of charge screening due to the presence of the electrolytes, which is expected to become stronger as the ion valency increases [34]. The salt addition establishes new interactions between the charged repeat units of the polymers with the small counterions. This effect of charge screening is responsible for the breaking of the poly-ion pairs to form poly-ion/counterion pairs. Such a phenomenon often leads to an increase in the hydration level of the PEC until the complete dissolution of their constituent poly-ions [16].

#### 3.2.2. Segregative Phase Separation

**Figure 5:**
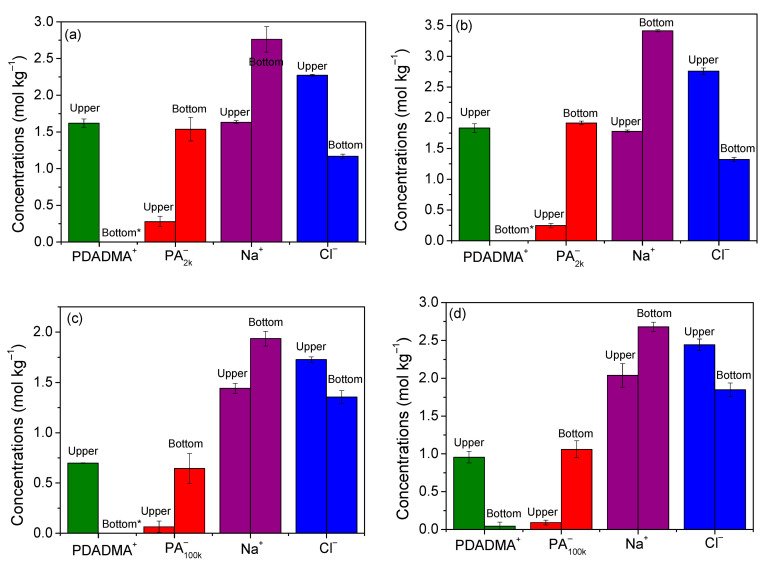
Distribution of poly-ions and small ions between the phases within the segregative phase separation region, expressed as mole of charges for poly-ions. The global compositions (wt %) are: (**a**) PDADMAPA_2k_: 17.2%; NaCl: 11.0%; water: 71.8%; (**b**) PDADMAPA_2k_: 21.0%; NaCl: 13.0%; water: 66.0%; (**c**) PDADMAPA_100k_: 7.0%; NaCl: 9.0%; water: 84.0%; and (**d**) PDADMAPA_100k_: 10.2%; NaCl: 12.0%; water: 77.8%. * Indicates PDADMA^+^ concentrations are too low to be determined.

The idea of polymer segregation at high salt concentrations comes from similar observations for systems containing polyelectrolytes and oppositely charged surfactants [27,35,36,37], and at least one system containing oppositely charged polyelectrolytes [38]. A more detailed investigation of this region was performed by analyzing the chemical composition in two systems for each PDADMAPA (see Figure 3). The concentrations of the ionic species are exhibited in Figure 5, and Table S1 in the Supporting Information presents a complete description of these systems. Both phases present a water content in the range of 65–82 wt %, meaning that these samples behave as aqueous two-phase systems essentially. Figure 5 and Table S1 also show that PDADMA^+^ concentrates entirely in the upper phase according to the analyses performed by TGA and mass balance. PA^−^, on the other hand, concentrates mostly in the bottom phase with a residual concentration in the upper phase. A previous study also reported the segregation between PA^−^ and the cationic surfactant dodecyltrimethylammonium aggregates at high salt concentrations [27]. That study showed that the surfactant is highly concentrated in the upper phase while the polyanion can be more concentrated in the bottom phase provided its molar mass is large enough. Those and the present results are qualitatively similar, confirming that the mixing between oppositely charged colloids tends to induce segregation at high salt concentrations. In addition, results in Figure 5 reveal that not only the poly-ions, but also the small ions display a significant segregation between the phases. Each small ion concentrates more in the phase enriched with its respective oppositely charged poly-ion.

The occurrence of this region resulted from the incompatibility of the polymers when they are mixed at high electrolyte concentrations. The point is to understand what contributions are relevant to determine the polymer incompatibility. However, the number of theoretical studies that try to explain this phase behavior is still limited. A liquid-state theory has successfully described the phase behavior of polyelectrolytes in simple salt solutions [39] and then it was also applied to mixtures of oppositely charged polyelectrolytes [32]. However, a complete description that captures both associative and segregative phase separation processes in oppositely charged polymers mixtures using this theory is still lacking.

A previous study by the work of Bergfeldt, Piculell and Linse investigated the conditions at which associative and segregative phase separation may occur, using the Flory–Huggins mean-field lattice model [40]. The authors considered the miscibility between the polymers and the solvent in terms of the Flory–Huggins interaction parameter, as well as the interaction parameter between the polymers. An overall trend is that the increase in the interaction parameter between the polymers makes the system change from an associative towards a segregative phase separation region with an intermediate homogeneous phase region. Although these results are qualitatively consistent, it is still difficult to apply them in a quantitative manner to the systems described in the present work. The difficulty is that the polyelectrolytes display different interaction parameters by varying the type of salt as well as the salt concentration. The polyelectrolytes used in this work, for example, when treated separately, present different phase behavior in salt solutions. Poly(acrylic acid) phase separates at lower ionization degrees in the presence of both NaCl and Na_2_SO_4_ [41,42]. Furthermore, a theoretical study suggests that the effect of PA^−^ ionization plays a role in determining its phase separation, showing that the phase separation no longer occurs above a certain ionization degree [41]. On the other hand, in the present investigation, we could not observe that any of these salts cause phase separation of PDADMA^+^ only. Furthermore, it has been reported that PDADMA^+^ precipitates only in the presence of the most chaotropic ions from the Hofmeister series, like I^−^, ClO_4_^−^, and SCN^−^ [12,43]. For this reason, we assume that the type of polymer and simple salt as well the pH play an important role in the segregation of these polymers when they are mixed.

We also observed an increased tendency towards segregative phase separation when the molar mass of PA^−^ is increased, with a larger biphase region for PDADMAPA_100k_ with NaCl than that for PDADMAPA_2k_ with the same salt. Similar to what is observed for the associative phase separation, the enlargement of the segregative phase separation region occurs because of the decrease in the translational and configurational entropy of the system due to the presence of larger polymer molecules.

In the present investigation, both associative and segregative phase separation regions were observed for the systems with NaCl while the mixtures with Na_2_SO_4_ exhibited only the associative phase separation. In order to confirm whether segregation would occur for the PDADMAPA complex with greater molar mass, some systems containing PDADMAPA_100k_ were mixed with Na_2_SO_4_ and no segregation was observed (see Figure S4 in the Supporting Information). A simple liquid-state theory investigation [39] showed that divalent co-ions such as SO_4_^2−^ are much less effective to induce phase separation of a polyanion than a monovalent co-ion. Considering the same number of anions (SO_4_^2−^ vs. Cl^−^), the number of Na^+^ ions in the systems with Na_2_SO_4_ is greater than that for the mixtures with NaCl. Although this work reports such a behavior for a single polyelectrolyte solution, we suppose that this increase in the small cation concentration turns the phase separation more difficult because of the greater translational entropy of the system.

## 4. Conclusions

This work describes the preparation of polyelectrolyte complexes free of small counterions. The approach previously proposed for surfactant and polymer mixtures was successfully applied, producing pure PECs displaying a very low residual chloride content. In addition, we confirmed that this methodology is associated with a limited extent of Hofmann elimination for the polycation. These results open up the possibility of extending studies towards more controlled experiments with PECs essentially free of their small counterions.

We have also presented results that provide a general picture of the effect of inorganic salts on the phase behavior of oppositely charged polymer mixtures beyond their associative phase separation. The chemical composition analyses in the second phase separation region shows that there is a strong segregation between PA^−^ and PDADMA^+^ in the presence of higher concentrations of NaCl. This phase separation region showed sensitivity to the molar mass of poly(acrylate) as well as to the chemical nature of the anion from the simple salts. Increasing molar polymer mass leads to a larger biphase region, while exchanging chloride by a divalent anion such as sulfate suppresses this macroscopic segregation. This behavior is discussed considering the contributions from configurational and translational entropy changes, the latter including the small ions.

The results also suggest that inorganic salts can be used as a simple way to isolate oppositely charged polyelectrolytes. In principle, we envisage that this method could be applied to biological systems, such as the coacervates that compose membraneless organelles. However, further studies must be performed to define which salts as well as the concentration and pH ranges provide a better separation of their macromolecular constituents. As this work points out, several factors could affect the segregation. For this reason, we also expect that further theoretical/computational modeling studies can also come out to shed light on these complex phase behaviors.

## Figures and Tables

**Table 1:** Extent of Hofmann elimination in PDADMAOH solutions as a function of the time after their preparation.

Equilibration Time at pH 13 (h)	Hofmann Elimination (mol%)
0	1.9 ± 0.8
24	8.3 ± 0.7
48	14 ± 5

**Table 2:** Molar ratio (n_Cl_^−^/n_Pol_^+^) between chloride and the positive charge units of PADADMA^+^, water content and water/unit charge pair molar ratio (n_H2O_/n_PEC_) for the PECs prepared through acid–base titration (Section 2.2). * PEC prepared through direct mixing (Section 2.6).

PEC	n_Cl_^−^/n_Pol_^+^	Water Content (wt %)	Molar Ratio (n_H2O_/n_PEC_)
PDADMAPA_2k_ *	0.130 ± 0.003	69.6 ± 0.1	26
PDADMAPA_2k_	0.006 ± 0.001	61.9 ± 0.4	18
PDADMAPA_100k_	0.002 ± 0.001	54.9 ± 0.1	13

## Data Availability

The data presented in this study are available in the article and Supporting information.

## Supplementary Materials

The following are available online at https://www.mdpi.com/article/10.3390/polym13142259/s1, Figure S1: pH titration curve of a poly(acrylate) solution with HCl solution, Figure S2: Thermogravimetric analysis of the upper phase of a mixture containing PDADMAPA2k, water and NaCl, Figure S3: Thermogravimetric analysis of the separated solutions of the polyelectrolytes used in this work under nitrogen atmosphere, Figure S4: Pseudo-ternary phase diagrams of mixtures containing PDADMAPA100k, water and salts assessed as an attempt to verify the occurrence of segregative phase separation, Table S1: Phase composition for the mixtures of NaCl, water and PDADMAPA in the segregative phase separation region.
