# Supplementary material for: Preparation of Poly(acrylate)/Poly(diallyldimethylammonium) Coacervates without Small Counterions and Their Phase Behavior upon Salt Addition towards Poly-Ions Segregation
Source: Polymers (Basel). 2021 Jul 9;13(14):2259. doi: 10.3390/polym13142259 (PMC8309331; doi:10.3390/polym13142259)
Supplement: Supplementary file 1 [file polymers-13-02259-s001.zip › polymers-1272928-supplementary.pdf]

polymers

Supporting Information

Preparation of Poly(acrylate)/Poly(diallyldimethylammonium) Coacervates without Small Counterions and their Phase Behavior upon Salts Addition towards Poly-ions Segregation

Marcos Vinícius Aquino Queirós and Watson Loh *

Institute of Chemistry, University of Campinas (UNICAMP); P. O. Box 6154, Campinas, SP 13083-970, Brazil; m212103@dac.unicamp.br (M.V.A.Q.)
* Correspondence: wloh@unicamp.br; Tel.: +55-19-35213148

1. Phase composition analyses in the segregative phase separation

1.1. Poly(acrylate) analysis

As described in the main text, the concentrations of the carboxylic groups from poly(acrylate) in each phase were determined by pH titrations. One example of these titrations presented in Figure S1. The charge concentration (C_{COO^-}/mol kg^{-1}) is calculated through Equation 1, where V_1 (L) and V_2 (L) stand for the volumes of acid required to achieve the first and second equivalence points, which are determined from the 1st derivative curve, C_{H^+} (mol L^{-1}) is the molar concentration of the acid, and m (kg) is the mass of the sample.

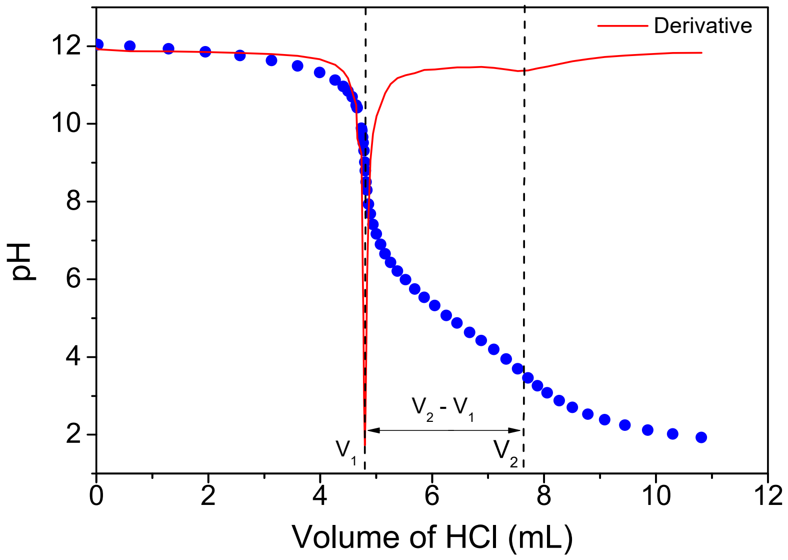

Figure S1. Typical pH titration curve with HCl solution for determination of poly(acrylate) content.

$$C_{coo^-} = [(V_2 - V_1) \times C_{H^+}]/m \quad (1)$$

1.2. Poly(diallyldimethylammonium) analysis

Figure S2 depicts a TGA curve used for the analysis of the upper phase of a mixture containing $PDADMAPA_{2k}$, water and NaCl. The first mass loss event corresponds to water evaporation. The second mass loss is attributed to the polymers thermal decomposition. TGA curves obtained for the pure polymers solutions (see Figure S3) indicate that their thermal decomposition occurs over the same temperature interval and, as such, PA^- and $PDADMA^+$ cannot be quantified independently with this technique. However, since PA^- content was independently determined by its acid-base titration (see above), the mass concentration of $PDADMA^+$ in the upper phase ($C_{PDADMA(Up)}$/g g^{-1}) can be determined according to Equation 2, where C_{pol} is the mass loss (wt %) from the second event and C_{PA-} (g g^{-1}) is the mass concentration of poly(acrylate) determined by pH titrations. The sum of the concentrations of all components determined by the different techniques for all of the phases analyzed results in a mass balance with a deviation lower than 3.5 wt %.

$$C_{PDADMA(Up)} = (C_{pol}/100) - C_{PA-} \quad (2)$$

We tried to use the same approach for the determination of the content of $PDADMA^+$ in the bottom phase but, because its content was rather low, inconsistent results were obtained from this mass balance. Therefore, Equation 3 was used to estimate the concentration of the polycation in the bottom phase ($C_{PDADMA(Bot)}$/g g^{-1}) by taking the mass balance between its concentration in the upper phase and its global amount ($m_{PDADMA(Tot)}$/g). In Equation 3, m_{Up} is the mass of the upper phase estimated by taking its volume and density (approximately 1.1 g cm^{-3} according to measurements of weighing and volume of the samples). However, even this estimation led to some values that are inconsistent (see Table S1), confirming that the concentrations of $PDADMA^+$ in the bottom phase are extremely low to be accurately determined through the techniques employed here.

$$C_{PDADMA(Bot)} = m_{PDADMA(Tot)} - C_{PDADMA(Up)} \times m_{Up} \quad (3)$$

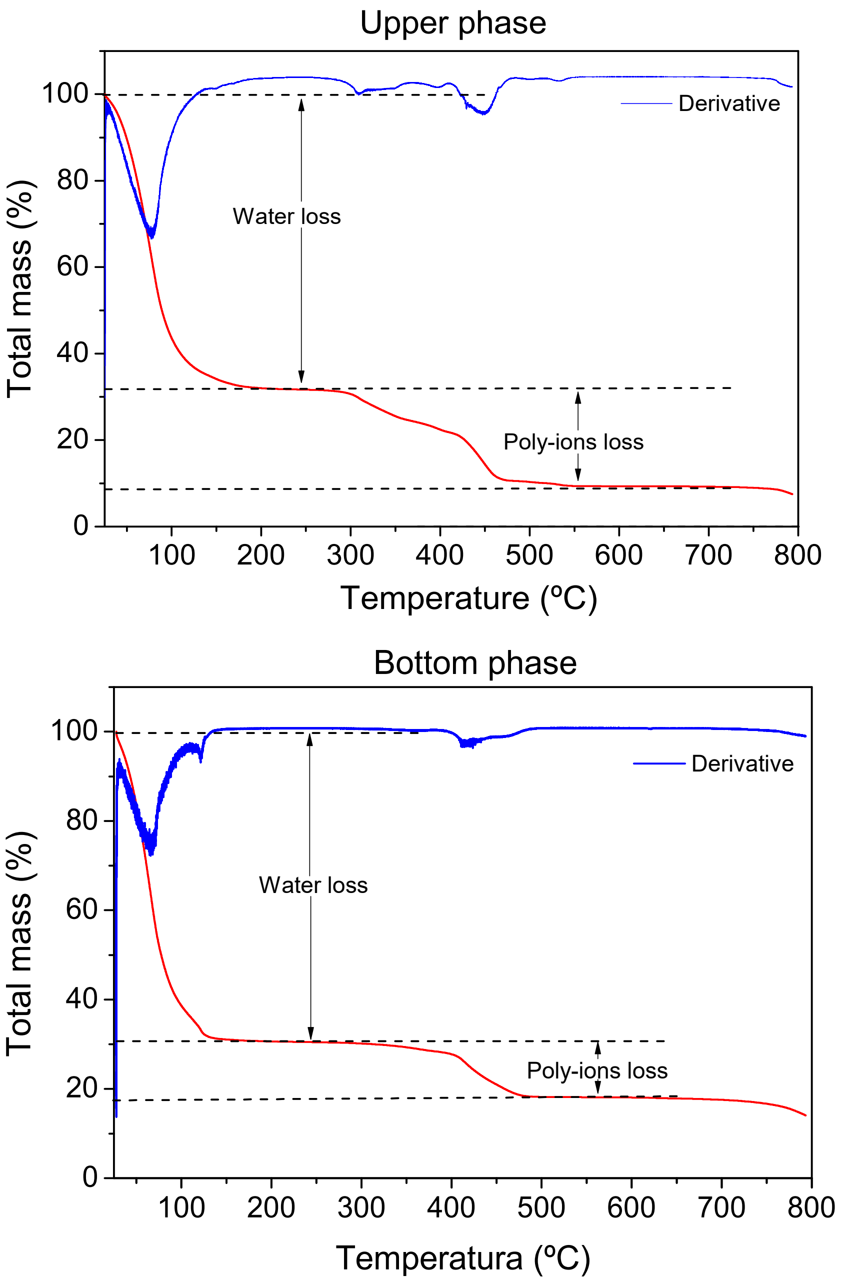

Figure S2. Thermogravimetric analysis of the upper and bottom phases of a mixture containing $PDADMAPA_{2k}$, water and NaCl.

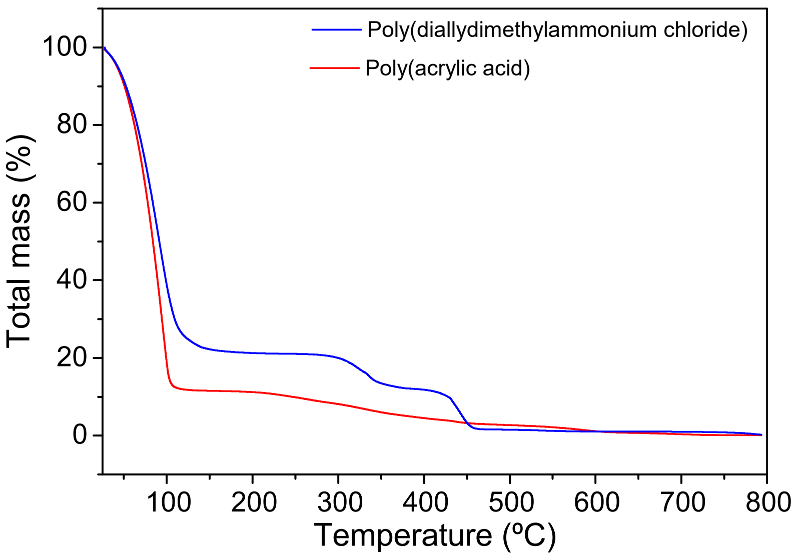

Figure S3. Thermogravimetric analysis of the separated solutions of the polyelectrolytes used in this work under nitrogen atmosphere. Poly(acrylic acid) 2k is shown in this analysis.

1.2 Mixtures containing PDADAMA, water and salts

In the manuscript we describe that a segregative phase separation region was observed for the mixture of $PDADAMAPA_{2k}$, water and NaCl, but not for the systems with Na_2SO_4. In Figure S4, we represent two experimental dilution lines that were investigated in mixtures of $PDAMAPA_{100k}$ with Na_2SO_4 in order to check for the occurrence of this phase segregation with a complex formed with PA of larger molar mass. This diagram shows that no segregation was observed for these mixtures at these two lines, hence, if this phenomenon occurs, it must be limited to a narrow region between these lines.

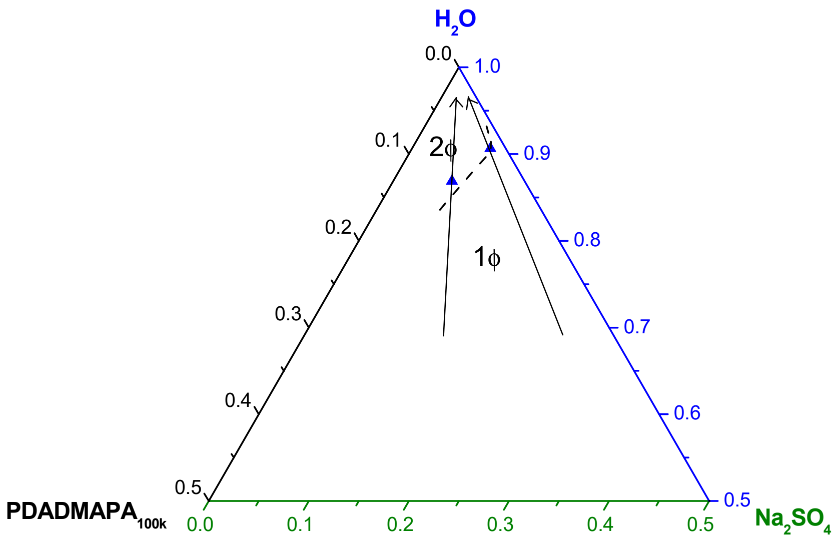

Figure S4. Pseudo-ternary phase diagrams of mixtures containing $PDADMAPA_{100k}$, water and salts assessed as an attempt to verify the occurrence of segregative phase separation. Experimental points (▲) are indicated for associative phase separation (tentatively represented by a dashed line), and the lines indicate the titrations performed.

Table S1. Phase compositions for the mixtures of NaCl, water and PDADMAPA in the segregative phase separation region. 2k and 100k represent the molar masses of the poly(acrylate). ND = not determined.

**Global composition (wt %)**	**NaCl**	11.0	13.0	9.0	12.0
	**Water**	71.8	66.0	84.0	77.8
	**PDADMAPA**	17.2 (2k)	21.0 (2k)	7.0 (100k)	10.2 (100k)
**PA^- (mol/kg)**	**Bottom phase**	1.5 ± 0.2	1.91 ± 0.03	0.6 ± 0.2	1.1 ± 0.1
	**Upper phase**	0.28 ± 0.07	0.25 ± 0.04	0.06 ± 0.06	0.09 ± 0.03
**$PDADMA^+$ (mol/kg)**	**Bottom phase**	< 0.02	< 0.08	ND	< 0.1
	**Upper phase**	1.62 ± 0.06	1.83 ± 0.07	0.70 ± 0.00	0.96 ± 0.08
**Cl^- (mol/kg)**	**Bottom phase**	1.17 ± 0.03	1.32 ± 0.03	1.35 ± 0.07	1.85 ± 0.09
	**Upper phase**	2.27 ± 0.01	2.76 ± 0.05	1.72 ± 0.03	2.44 ± 0.07
**Na^+ (mol/kg)**	**Bottom phase**	2.76 ± 0.17	3.41 ± 0.02	1.94 ± 0.07	2.68 ± 0.06
	**Upper phase**	1.63 ± 0.02	1.78 ± 0.02	1.44 ± 0.05	2.04 ± 0.16
**Water (wt %)**	**Bottom phase**	69.4 ± 0.2	67.4 ± 0.1	78.2 ± 2.7	73.3 ± 1.6
	**Upper phase**	68.0 ± 0.0	64.8 ± 0.9	81.9 ± 0.6	75.8 ± 1.0
